# Association of volatile anesthesia exposure and depth with emergence agitation and delirium in children: Prospective observational cohort study

**DOI:** 10.3389/fped.2023.1115124

**Published:** 2023-03-23

**Authors:** Yinan Zhang, Qiuying Zhang, Shan Xu, Xiaoxi Zhang, Wenxu Gao, Yu Chen, Zhaoqiong Zhu

**Affiliations:** ^1^ Department of Anesthesiology, Affiliated Hospital of Zunyi Medical University, Zunyi, China; ^2^ Translational Neurology Laboratory, Affiliated Hospital of ZunYi Medical University, Zunyi, China

**Keywords:** emergence agitation, sevoflurane anesthesia, children, anesthesia exposure, depth of anesthesia, emergence delirium

## Abstract

**Background:**

Sevoflurane anesthesia is widely used in pediatric ambulatory surgery. However, emergency agitation (EA) and emergency delirium (ED), as major complications following sevoflurane anesthesia in children, pose risks to surgery and prognosis. Identifying the high risk of EA/ED, especially anesthesia exposure and the depth of anesthesia, may allow preemptive treatment.

**Methods:**

A total of 137 patients were prospectively enrolled in this single-center observational cohort study to assess the incidence of EA or ED. Multivariable logistic regression analyses were used to test the association between volatile anesthesia exposure and depth with EA or ED. The Richmond Agitation and Sedation Scale (RASS), Pediatric Anesthesia Emergence Delirium Scale (PAED) and Face, Legs, Activity, Cry, and Consolability (FLACC) behavioural pain scale was used to assess the severity of EA or ED severity and pain. Bispectral index (BIS) to monitor the depth of anesthesia, as well as Time_LOW−BIS_/Time_ANES_ %, EtSevo (%) and EtSevo-time AUC were included in the multivariate logistic regression model as independent variables to analyze their association with EA or ED.

**Results:**

The overall prevalence of EA and ED was 73/137 (53.3%) and 75/137 (54.7%) respectively, where 48/137 (35.0%), 19/137 (13.9%), and 6/137 (4.4%) had mild, moderate, and severe EA. When the recovery period was lengthened, the prevalence of ED and extent of FLACC decreased and finally normalized within 30 min in recovered period. Multivariable logistic regression demonstrated that intraoperative agitation [2.84 (1.08, 7.47) *p* = 0.034], peak FLACC [2.56 (1.70, 3.85) *p *< 0.001] and adverse event (respiratory complications) [0.03 (0.00, 0.29) *p* = 0.003] were independently associated with higher odds of EA. Taking EtSevo-time AUC ≤ 2,000 as a reference, the incidence of EA were [15.84 (2.15, 116.98) *p *= 0.002] times and 16.59 (2.42, 113.83) *p *= 0.009] times for EtSevo-time AUC 2,500–3,000 and EtSevo-time AUC > 3,000, respectively. Peak FLACC [3.46 (2.13, 5.62) *p* < 0.001] and intraoperative agitation [5.61 (1.99, 15.86) *p *= 0.001] were independently associated with higher odds of developing ED. EtSevo (%), intraoperative BIS value and the percentage of the duration of anesthesia at different depths of anesthesia (BIS ≤ 40, BIS ≤ 30, BIS ≤ 20) were not associated with EA and ED.

**Conclusions:**

For pediatrics undergoing ambulatory surgery where sevoflurane anesthesia was administered, EA was associated with surgical time, peak FLACC, respiratory complications, and “EtSevo-time AUC” with a dose-response relationship; ED was associated with peak FLACC and intraoperative agitation.

## Introduction

Sevoflurane anesthesia has advantages of rapid uptake and elimination and shows reduced opioid adverse events in pediatric ambulatory surgery (1–3). However, emergence agitation (EA) and emergence delirium (ED) are recognized as a significant complication after sevoflurane anesthesia in pediatric patients, with a reported prevalence between 10% and 80% (4–6). There's a difference between EA and ED. EA is commonly considered not always associated with significant changes in behavior or cognition (6). Intensity of EA can be scored using tools such as the Richmond Agitation-Sedation Scale (RASS), it is the version of typical sedation scales (6). Unlike EA, ED is acute brain dysfunction, which occurs in the setting of systemic disease or derangement (6). The Pediatric Anesthesia Emergence Delirium (PAED) is considered an effective tool for ED assessment and is widely used in the post-anesthesia care unit (PACU) (5). Transient agitation/delirium from sevoflurane anesthesia can lead to a variety of adverse events, such as airway spasm, shedding or displaced tracheal tube, dehiscence, or bleeding, resulting in serious secondary complications, longer PACU stays, and delayed discharge. The fundamental cause of the disorder remains unknown.

Several perioperative factors may be associated with EA or ED. Volatile anesthesia exposure and depth are the major risk factors for EA/ED (7). Volatile agents induce EA/ED nearly four times more often than intravenous anesthetics, particular sevoflurane (4). A prospective observational study of approximately 2,000 patients reported that volatile anesthetic agents were associated with a higher occurrence rate of EA (8). In a 2018 randomized clinical trial (RCT), sevoflurane was reported to increase the risk of EA by more than 17-fold after nasal surgery (9).

The bispectral index (BIS) monitor is a common method for monitoring the depth of anesthesia (10, 11). Previous studies have suggested low BIS value (<40) affects postoperative recovery, including postoperative delirium, mortality (12–14). However, a RCT of sevoflurane in children suggested there was no significant effect of BIS-guided deep vs. light anesthesia on severe EA (15). A definitive relationship between low BIS and EA/ED remains inconclusive (16).

To explore the relationship between depth of anesthesia or volatile exposure and EA or ED, we conducted a prospective observational cohort study that explored independent association between sevoflurane exposure and low BIS with EA/ED in children with sevoflurane anesthesia undergoing ambulatory surgery. We also explored the utility of the age, preoperative modified Yale Preoperative Anxiety Scale (m-YPAS), anesthesia time, surgical time, the post-operative peak scores of Face, Legs, Activity, Cry, and Consolability (peak FLACC), intraoperative agitation and adverse event (respiratory complications) for EA or ED prediction.

## Methods

### Study design and ethical considerations

This present study was conducted at a single institution (Affiliated Hospital of Zunyi Medical University, China, a 2800-bed academic center). The study was approved by the Affiliated Hospital of Zunyi Medical University Biomedical Research Ethics Committees (reference number: KLL-2022-623) and registered with the Chinese Clinical Trial Registry (ChiCTR2200062680). Recruitment occurred between August 15 and October 15, 2022. Written informed consent was obtained from child's parents. This study was consistent with the Strengthening the Reporting of Observational Studies in Epidemiology (STROBE) reporting guidelines (17).

### Population

The inclusion criteria were as follows: Children aged 4–12 years scheduled for same-day or ambulatory circumcision treatment under general anesthesia and American Society of Anesthesiology (ASA) physical status I–II. Exclusion criteria were developmental delay or neurologic impairment; autism spectrum disorder (ASD) or reactive attachment disorder (RAD); premedication interfering with the central nervous system; history of allergies, or adverse reactions to intraoperative medications. The diagnoses of exclusion were verified by the description of the children's parents, or diagnosis by a paediatrician. Developmental delay is defined as significantly delays in cognitive and physical development of children, including motor adaptive skills, language or personal and social behavior (18). Neurologic impairment was defined as functional and/or intellectual impairment resulting from a neurologic disease. Premedication interfering with the central nervous system refers to children who received propofol, morphine, fentanyl, midazolam, ketamine, and dexmedetomidine within 24 h before the trial.

### Study protocol

#### Process of anesthesia and operation

After screening for eligibility, informed consent for processing of personal data from the guardian was obtained at the time of the patient's appointment for surgery. On the day of surgery, after separation from the parents, the patients were assessed for preoperative anxiety by the m-YPAS (15) in the operating room.

Volatile induction and maintenance anesthesia (VIMA) was performed *via* inhalation of sevoflurane through a mask with the patient's consent and cooperation. A dorsal penile nerve block with 0.25% ropivacaine and 1% lidocaine compound (0.15 ml/kg) was administered following induction. Two pediatric urologists on the same team performed the circumcision (sutureless prepuceplasty). Volatile dose, airway management, management for any arising complications were administered at the discretion of the anesthesiologist.

After completion of the procedure, the patient was transferred to PACU for recovery and assessment of the level of agitation and delirium, the full recovery routine usually lasts 30 min in PACU and the patients are returned to the pediatric day ward when awake. Patients were discharged when the Modified Aldrete Score was ≥9 (19).

#### Data acquisition

• Pre-operation: age, height, weight, body mass index (BMI), and ASA class. Preoperative anxiety was assessed using the m-YPAS after separation from the parents, which included four items: activity, emotional expressivity, state of arousal, vocalization.
• Surgical data included the type of procedure and duration of surgery (from incision to the end of surgery).
• Anesthesia data: routine monitoring included noninvasive blood pressure (NBP), heart rate (HR), pulse oximetry (SpO_2_), electrocardiogram (ECG), end-tidal sevoflurane concentration (EtSevo) with once every 15 s, temperature, end-tidal CO_2_ concentration (EtCO_2_). Other data included anesthesia duration (from beginning of volatilization to removal of the mask), intraoperative agitation (nonpurposeful movement during anesthesia), and respiratory complications such as upper airway obstruction, hypoxia, bronchospasm, and cough, which were recorded in operation room and PACU.
• BIS: The depth of sedation was monitored by BIS monitoring. The BIS sensor remained adhered to the forehead, which had been cleaned with alcohol. The data were recorded and obtained every 15 s from pre-anesthesia to the end of anesthesia.
• Emergence and recovery period data: emergence period is defined as the episode from removal of the mask to full recovery, where full recovery is defined as the patient's Richmond Agitation and Sedation Scale (RASS) −1 or 0 (sustained alert and calmer sustained awakening, with eye contact, to voice, for ≥10 s) (20). The recovery period is defined as the episode from full recovery to leaving the PACU. EA during the emergence period and ED during the recovery period were assessed. The RASS reached −1 or 0 as the primary time-point to differentiate ED from EA.

#### Emergency agitation

The RASS is recognized as the most commonly used sedation and agitation assessment tool in the PACU or intensive care unit (ICU) (20). We assessed the level of EA through RASS during the emergence period because the patient might still be drowsy due to residual sevoflurane in the system. During the emergence period, agitation was assessed by the anesthesiologist every minute, and patients were considered agitated once their RASS score reached 1–4 on any of the assessments. Patients were classified into three groups based on RASS scores. Mild: RASS +1 and lasting <3 min; Moderate: RASS +1 and lasting ≥3 min or RASS +2 and lasting <3 min; Severe: RASS +2 and lasting ≥3 min or RASS ≥+3.

#### Emergency delirium

The PAED (20) and FLACC (15) were used to assess the level of delirium and pain at different time points, including instantly upon recovery (RASS reach −1 or 0), every 1 min for the first 5 min of the recovery period, and every 5 min thereafter, until the patient was mentally alert in the PACU. The peak PAED score was extracted, the children were considered to have ED when PAED ≥ 10, according to a previous study (15).

### Statistical analysis

#### Derived data

We derived the acquisitive data and calculated Time_LOW−BIS_/Time_ANES_ % and EtSevo-time AUC. BIS data and EtSevo(%) were fitted to the exposure duration to further detected the association between depth of anesthesia or sevoflurane exposure and EA/ED.

• Time_LOW−BIS_/Time_ANES_ %
  Time_LOW−BIS_/Time_ANES_ %included Time_BIS ≤ 40_/Time_ANES_ %, Time_BIS ≤ 30_/Time_ANES_ % and Time_BIS ≤ 20_/Time_ANES_ %, which were respectively expressed as the percentage of the duration of BIS ≤ 40, BIS ≤ 30, BIS ≤ 20 in the total anesthesia duration.
• EtSevo-time AUC
  To better fit sevoflurane concentration and anesthesia duration, we used the area under curve (AUC), which is a pharmacokinetic parameter and represents the plasma drug concentration-time curve. Sevoflurane concentrations were extracted at 5 min intervals during anesthetic exposure as the drug concentration at discrete points in time, using GraphPad PRISM software to calculate definite integral of the concentration of EtSevo as a function of exposure time with time-point as the *X*-axis and EtSevo (%) as the *Y*-axis (21). This reflects actual body exposure to sevoflurane after administration.

#### Sample size and statistical analysis

Based on sample size estimates from previous literature, the prevalence of EA or ED in children under sevoflurane anesthesia is approximately 60%–70% (5, 22). Group sample sizes of 114 patients were required to reach a margin of error of 0.1 for 90.144% confidence interval (CI). A two-sided *Z* test with unpooled variance is used to test the statistics. The significance level of the test was set at *p *< 0.05. The final target of evaluable cases was 137 to account for a 20% attrition rate related to protocol violations.

Continuous variables were summarized by medians and interquartile ranges due to nonnormality. Categorical variables were expressed as counts and percentages. The primary outcomes were EA and ED. Wilcoxon rank sum test, chi-square test, or Fisher exact test, as appropriate, were used compared the differences between continuous or categorical variables and between the two outcome indicators.

A univariate logistic regression model was established to calculate the crude odds ratio (OR) of each variable. Two multivariable logistic regression models were performed to assess the association between EA or ED and 6 independent variables: EtSevo-time AUC, BIS, surgical time, peak FLACC, intraoperative agitation, adverse event (respiratory complications). The variance inflation factor (VIF) measures the degree of multicollinearity or collinearity in the regression model, *p *< 0.05 was statistically significant. We used IBM SPSS Statistics, version 25 (IBM Corp) and GraphPad PRISM (GraphPad Software) for statistical analyses.

## Results

**Figure 1:**
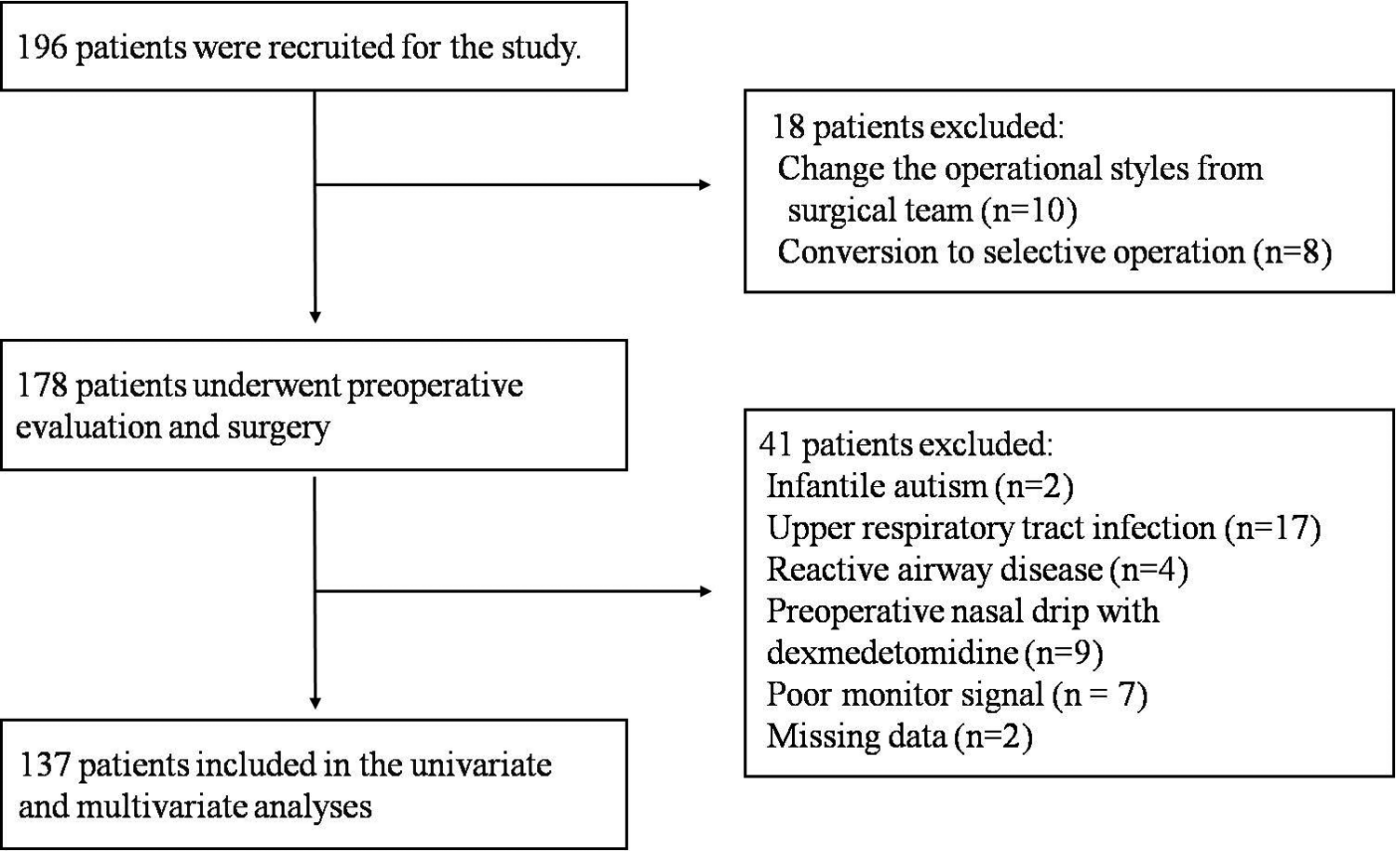
Patient flow diagram showing the number of patients included and excluded.

A total of 196 patients were recruited between August 15 and October15, 2022. We excluded 73 patients because they did not complete day-surgery and observations, or had events that might interfere with EA and ED. Ultimately a total of 137 patients were included in the analyses (Figure 1).

The clinical characteristics of the included patients are summarized in Tables 1, 2, including patient demographics, BIS scores and characteristics, sevoflurane exposure parameters, intraoperative events, and EA/ED outcomes. Patients were ASA Physical Status I or II (100%), the median of age, BMI and pre-operation m-YPAS were 9.0 (8.0, 10.0) years, 16.5 (14.7, 20.3) kg/m^2^ and 29.2 (22.9, 35.4). The median BIS value was 38.6 (35.0, 43.0), and BIS > 40 and BIS ≤ 40 were 57/137 (41.6%) and 80/137 (58.4%), respectively. The percentage of the duration of BIS ≤ 40, BIS ≤ 30, BIS ≤ 20 in the total anesthesia duration were 84.7% (60.4%, 97.1%), 35.3% (16.8%, 60.4%), 9.4% (2.6%, 17.6%), respectively. The median EtSevo and EtSevo-time AUC were 4.4% (4.0%, 4.9%) and 2,618.0 (2,349.5, 3,066.0), and the EtSevo-time AUC was calculated using software (Supplementary Figure S1).

**Table 1:** Baseline and clinical characteristics of the included patients (*n* = 137).

Variables	Values
Age (year)	9.0 (8.0, 10,0)
Weight (kg)	27.33 (24.2, 63.1)
BMI (kg/m^2^)	16.5 (14.7, 20.3)
**ASA physical status, *N* (%)**	
I–II	137/137 (100%)
III–IV	0/137 (0%)
m-YPAS	29.2 (22.9, 35.4)
BIS_value_	38.6 (35.0, 43.0)
**BIS_proportion_, *N* (%)**	
BIS > 40 (%)	57/137 (41.6%)
BIS ≤ 40 (%)	80/137 (58.4%)
**TimeLOW-BIS/Time_ANES_%, *N* (%)**	
TimeBIS ≤ 40/Time_ANES_%	84.7% (60.4%, 97.1%)
TimeBIS ≤ 30/Time_ANES_%	35.3% (16.8%, 60.4%)
TimeBIS ≤ 20/Time_ANES_%	9.4% (2.6%, 17.6%)
EtSevo (%)	4.4 (4.0, 4.9)
EtSevo-time AUC	2,618.0 (2,349.5, 3,066.0)
Anesthesia time, (min)	9.0 (8.0, 11.0)
Surgical time, (min)	5.0 (5.0, 6.0)
Peak FLACC	3.0 (1.0, 3.0)
**Intraoperative agitation, *N* (%)**	
No	70/137 (51.1%)
Yes	67/137 (48.9%)
**Adverse event (respiratory complications), *N* (%)**	
No	121/137 (88.3%)
Yes	16/137 (11.7%)

Values are presented as Median (25%, 75% Quartile), *N* (%). BMI, body mass index; ASA, American Society of Anesthesiologists; m-YPAS, modified Yale preoperative anxiety scale; BIS, bispectral index; EtSevo, end-tidal sevoflurane concentration; AUC, the area under curve; Peak FLACC, the peak scores of face, legs, activity, cry, and consolability.

**Table 2:** Patient outcomes during emergence and recovered period.

Characteristics	Values								
**Emergence agitation (emergency period)**									
Total, *N* (%)	73/137 (53.3%)								
Mild, *N* (%)	48/137 (35.0%)								
Moderate, *N* (%)	19/137 (13.9%)								
Severe, *N* (%)	6/137 (4.4%)								
Duration (min)	1 (0.0, 2.0)								
**Emergence delirium (recovered period and RASS is −1 or 0)**									
Total, *N* (%)	75/137 (54.7%)								
Peak FLACC	3.0 (1.0, 3.0)								
Peak PAED	10.0 (8.0, 12.0)								
**Characteristics of emergence delirium over time**									
	0 min	1 min	2 min	4 min	6 min	8 min	10 min	15 min	Discharge
Occurrence of ED, *N* (%)	77/137 (56.20%)	26/137 (18.98%)	10/137 (7.30%)	10/137 (7.30%)	6/137 (4.38%)	2/137 (1.46%)	2/137 (1.46%)	0/137 (0.00%)	0/137 (0.00%)
PAED	10 (8, 12)	3 (0, 8)	0 (0, 0)	0 (0, 0)	0 (0, 0)	0 (0, 0)	0 (0, 0)	0 (0, 0)	0 (0, 0)
FLACC	3 (2, 6)	0 (0, 3)	0 (0, 0)	0 (0, 0)	0 (0, 0)	0 (0, 0)	0 (0, 0)	0 (0, 0)	0 (0, 0)
**Both emergence agitation and emergence delirium**									
Total, *N* (%)	59/137 (43.1%)								

Values are presented as median (25%, 75% quartile), *N* (%). Abbreviations: Peak FLACC, the peak scores of face, legs, activity, cry, and consolability; Peak PAED, the peak scores of pediatric anesthesia emergence delirium scale; Discharge, discharge from post-anesthesia care unit. Mild: RASS +1 and lasting <3 min; Moderate: RASS +1 and lasting ≥3 min or RASS +2 and lasting <3 min; Severe: RASS +2 and lasting ≥3 min or RASS ≥+2.

The overall prevalence rates of EA and ED were 73/137 (53.3%) and 75/137 (54.7%) respectively, with both EA and ED occurring in 59/137(43.1%) patients. The observed prevalence of different grades of EA was mild 48/137 (35.0%), moderate 19/137 (13.9%), and severe 6/137 (4.4%), and the median agitation duration was 1 (0.0, 2.0) min. Patients displaying a severe grade and frequent agitation lasting ≥3 min were less frequent. The prevalence of ED and assessment of FLACC decreased when the recovery period was prolonged, and finally disappeared within 30 min in the recovered period.

Based on univariate analysis, EtSevo-time AUC, intraoperative agitation, surgical time and post-operative peak FLACC were associated with EA; unadjusted *p *< 0.05. Intraoperative agitation and peak FLACC were associated with ED. EtSevo, intraoperative BIS value and the percentage of the duration for different depths of anesthesia (BIS ≤ 40, BIS ≤ 30, BIS ≤ 20) in the total duration of anesthesia were not associated with EA and ED. We made two univariate logistic regression models (Supplementary Tables S1, S2). The outcomes were same as univariate analysis. We established two multivariable logistics models to evaluate the independent association between 6 variables and EA (Table 3) and ED (Table 4) [odds ratio; 95% CI; adjusted *p*]: intraoperative agitation [2.84 (1.08, 7.47) *p *= 0.034], peak FLACC[2.56 (1.70, 3.85) *p *<* *0.001] and adverse event (respiratory complications) [0.03 (0.00, 0.29) *p *= 0.003] were independently associated with higher odds of EA. Taking the EtSevo-time AUC ≤ 2,000 as a reference, the incidence of EA were [15.84 (2.15, 116.98) *p *= 0.002] times and 16.59 (2.42, 113.83) *p *= 0.009] times for EtSevo-time AUC 2,500–3,000 and EtSevo-time AUC > 3,000, respectively (Table 5). Peak FLACC [3.46 (2.13, 5.62) *p *< 0.001] and intraoperative agitation [5.61 (1.99, 15.86) *p *= 0.001] were independently associated with higher odds of ED.

**Table 3:** Multivariable logistic regression model for emergence agitation.

Variable		Emergence Agitation	No Emergence Agitation	Odds Ratio (95%CI)	Adjusted *p* value
EtSevo-timeAUC, *N* (%)	≤2,000	2 (20.0%)	8 (80.0%)	Reference	
	2,000–2,500	20 (43.5%)	26 (56.5%)	3.99 (0.59, 26.91)	0.155
	2,500–3,000	19 (54.3%)	16 (45.7%)	15.84 (2.15, 116.98)	0.007
	>3,000	32 (69.6%)	14 (30.4%)	16.59 (2.42, 113.83)	0.004
BIS, *N* (%)	BIS > 40	29 (50.9%)	28 (49.1%)	Reference	
	BIS ≤ 40	44 (55.0%)	36 (45.0%)	2.39 (0.88, 6.47)	0.086
Surgical time (min)		5.00 (5.00, 6.00)	5.00 (4.00, 5.00)	1.48 (0.96, 2.26)	0.074
Peak FLACC, *N* (%)		3.00 (3.00, 5.00)	1.50 (1.00, 3.00)	2.56 (1.70, 3.85)	0.000
Intraoperative agitation, *N* (%)	No	24 (37.5%)	40 (62.5%)	Reference	
	Yes	43 (58.9%)	30 (41.1%)	2.84 (1.08, 7.47)	0.034
Adverse event (respiratory complications), *N* (%)	No	9 (14.1%)	55 (85.9%)	Reference	
	Yes	7 (9.6%)	66 (90.4%)	0.03 (0.00, 0.29)	0.003

Values are presented as Median (25%, 75% Quartile), *N* (%). EtSevo, end-tidal sevoflurane concentration; AUC, the area under curve; Peak FLACC, the peak scores of face, legs, activity, cry, and consolability.

**Table 4:** Multivariable logistic regression model for emergence delirium.

Variable		ED	No ED	Odds Ratio (95%CI)	Adjusted *p* value
EtSevo-timeAUC, *N* (%)	≤2,000	2 (20.0%)	8 (80.0%)	Reference	
	2,000–2,500	20 (43.5%)	26 (56.5%)	1.30 (0.26, 6.52)	0.750
	2,500–3,000	22 (64.7%)	12 (35.3%)	1.49 (0.28, 7.75)	0.639
	>3,000	29 (61.7%)	18 (38.3%)	1.61 (0.31, 8.16)	0.566
BIS, *N* (%)	BIS > 40	37 (59.7%)	25 (40.3%)	Reference	
	BIS ≤ 40	43 (57.3%)	32 (42.7%)	1.85 (0.74, 4.63)	0.189
Surgical time (min)		5.00 (5.00, 6.00)	5.00 (5.00, 6.00)	0.95 (0.70, 1.30)	0.759
Peak FLACC, *N* (%)		3.00 (3.00, 5.00)	1.50 (1.00, 3.00)	3.23 (2.02, 5.16)	0.000
Intraoperative agitation, *N* (%)	No	21 (33.9%)	41 (66.1%)	Reference	
	Yes	46 (61.3%)	28 (38.7%)	3.78 (1.52, 9.44)	0.004
Adverse event (Respiratory complications), *N* (%)	No	4 (6.5%)	58 (93.5%)	Reference	
	Yes	12 (16.0%)	63 (84.0%)	1.33 (0.26, 6.79)	0.733

Values are presented as Median (25%, 75% Quartile), *N* (%). EtSevo, end-tidal sevoflurane concentration; AUC, the area under curve; Peak FLACC, the peak scores of face, legs, activity, cry, and consolability.

**Table 5:** Risk of emergence agitation for different EtSevo-time AUC range.

EtSevo-time AUC	Emergence agitation *N* (%)	No emergence agitation *N* (%)	Odds Ratio (95%CI)		Adjusted *p* value
>3,000	32 (69.6%)	14 (30.4%)	16.59 (2.42, 113.83)	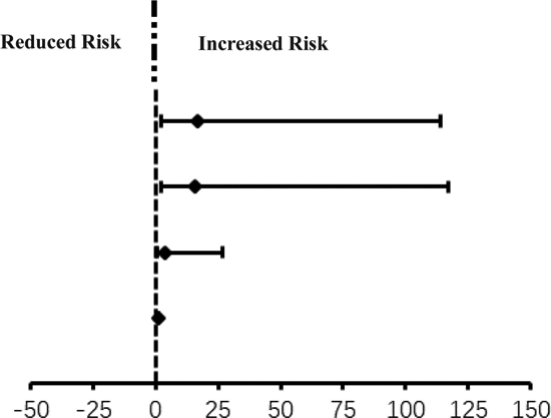	0.009
2,500–3,000	19 (54.3%)	16 (45.7%)	15.84 (2.15, 116.98)		0.002
2,000–2,500	20 (43.5%)	26 (56.5%)	3.99 (0.59, 26.91)		0.168
≤2,000	2 (20.0%)	8 (80.0%)	1 (1, 1)		1 (reference)

Values are presented as Median (25%, 75% Quartile), *N* (%). EtSevo, end-tidal sevoflurane concentration; AUC, the area under curve.

## Discussion

In an observational cohort study of pediatric patients who underwent ambulatory surgery under volatile anesthesia, we found that (1) EtSevo-time AUC fitted by EtSevo (%) and exposure time is independent risk factor for EA. Further it was found the risk factors had a dose-dependent effect, but not the two parameters alone. (2) Using BIS to assess the depth of anesthesia, it was found that there was no correlation with EA/ED. Similarly duration of low-normal anesthesia had not correlation with the occurrence of EA/ED (3) only 4.4% patients had severe EA/ED, and the median PAED score fell from 10 to 0 within 30 min of recover from anesthesia.

In this study, we found that EtSevo-time AUC formed by fitting EtSevo (%) and exposure time was the main factor influencing EA, although EtSevo (%) and exposure time were not the independent factor. Meanwhile, EtSevo-time AUC trend to have a continuous dose-dependent effect on EA risk. AUC is recommended as the primary measurement with drug absorption data by The Food and Drug Administration (FDA) (23), and is used in many research fields to assess certain levels of exposures, such as pharmacokinetics, chemistry, nutrition science, physics (24, 25). In this study, we used GraphPad PRISM software to calculate EtSevo-time AUC, which is the definite integral of the concentration of EtSevo as a function of exposure time, and represented the total drug exposure. The use of AUC to assess levels of drug exposures during anesthesia is still tentative, and more studies are needed to confirm this.

The incidence of EA in patients in the prospective cohort was 20.0% with EtSevo-time AUC ≤ 2,000, 43.5% with EtSevo-time AUC 2,000–2,500, 54.3% with EtSevo-time AUC 2,500–3,000, 69.6% with EtSevo-time AUC > 3,000. It is suggested that anesthesiologists should pay attention to concentration of sevoflurane as well as exposure time. Excessive exposure may increase the accumulation of compounds in the body, which are more difficult to cleared during emergence (26, 27), resulting in an imbalance in the rate of recovery for arousal state and content of consciousness, referred to as the theory of differential clearance for EA or ED (28, 29). Additional studies are needed to validate the correlation between EtSevo-time AUC and EA, which may be a new indicator for monitoring sevoflurane during anesthesia.

The effect of depth of anesthesia on ED remains uncertain. In the study, we obtained the BIS value every 15 s during anesthesia for each patient, and found that the median BIS was not associated with EA or ED. We further found that the percentage of the duration of BIS ≤ 40, BIS ≤ 30, BIS ≤ 20 in the total anesthesia time was no correlation with EA or ED. This suggested that neither the depth of anesthesia nor the duration of low-normal anesthesia is an independent risk factor for EA or ED with transient exposure to sevoflurane. This result is similar to previous studies (15, 30). Indeed, the BIS represents the level of consciousness under anesthesia, which has proven controversial among anesthesiologists (31, 32). Intraoperative isoelectric electroencephalography (EEG) has been considered to be associated with delirium in many studies (33, 34), however, two recent studies of isoelectric EEG in toddlers or older adults showed that it was not independently correlated with ED (35, 36). More high-quality RCT trials are needed to confirm BIS- or EEG-guided anesthesia.

In this study, we distinguished between EA and ED. EA is a common symptom during recovery from general anesthesia and has been described as an unpleasant state of extreme arousal that emergence alone and does not always follow delirium. ED is defined as acute brain dysfunction accompanied by mental confusion, agitation, and disinhibition during recovery from general anesthesia (37). The PAED scale assesses for the Diagnostic and Statistical Manual of Mental Disorders (DSM) criteria for delirium, including eye contact with the caregiver, unawareness of surroundings, decrease in purposeful actions and restlessness or inconsolability, which is applicable to children after recovery from anesthesia (6). In this study, patients who sustained awakening with eye contact or voice for ≥10 s (RASS ≥ −1) were assessed using the PAED scale. In contrast, patients with RASS < −1 could not accurately assess the four indicators of PAED because they were unconsciousness. On the other hand, we used the RASS to assess the occurrence of EA during sedation with anesthetic to consciousness. A study on agitation in children also suggested that the evaluation of agitation using the RASS score at emergence from anesthesia is useful to predicting the occurrence of agitation in the recovery phase (20). It is important to distinguish between EA and ED with similar symptoms and behaviors to study their epidemiology and provide appropriate treatment.

This study showed that children undergoing ambulatory surgery with sevoflurane anesthesia had a high incidence of EA or ED, but most had mild to moderate agitation, and only 4.4% had severe agitation. Delirium occurred within 30 min of recover from anesthesia and returned to normal before PACU discharge (within 30 min after recovery), consisting with other similar studies (15).

The risk factors for EA/ED are presented in three major categories: patient-related, anesthesia-related, and surgical factors. We collected data on preoperative anxious, surgical duration, peak value of FLACC, and respiratory complications (hypoxemia, spasm, respiratory depression), the result shows that preoperative anxious, surgical duration were not associated with EA or ED. The independent risk factors for EA and ED were peak value of FLACC, intraoperative agitation and respiratory complications. Postoperative pain and intraoperative agitation have been clearly identified as risk factor for EA or ED. When the analgesic effect was poor, the rates of EA and ED increased by 1.86 times and 2.46 times (38). In this study, we observed that some patients experienced restlessness or agitation when the nerve block was administered after induction. Once the block was complete, the agitation will disappear. These results are consistent with previous studies (20, 39), and they deserve attention from anesthesiologists.

### Limitations

This study has some limitations. Firstly, the single center of the study, limitation to one surgical procedure, and anesthetic method in this study only represented the observation results for pediatric ambulatory surgery undergoing sevoflurane anesthesia. Due to this limited sample, the EA- or ED-related findings are not directly translatable to the general population. Multi-center trials might refine the interpretation of this trial. Secondly, the BIS data, which measures the level of consciousness under anesthesia, has proved controversial. This study should be repeated with other assessments of the depth of anesthesia. Thirdly, the psychiatric symptoms of children after discharge were not described. Fourthly, we did not assess the impact of hypoactive delirium on outcomes following general anesthesia, this aspects needs more attention.

## Conclusions

We found that using sevoflurane anesthesia in pediatric patients undergoing ambulatory surgery, the incidence of EA was associated with the fitted value “EtSevo-time AUC”, with a dose-related response, but not with depth of anesthesia as assessed by BIS and the duration of low-normal anesthesia. The fit of inhalation concentration and duration in sevoflurane anesthesia is a better indicator of anesthesia exposure than a single element. Further studies are warranted to validate our results and gain insight into the possible explanations for such an association.

## Data Availability

The original contributions presented in the study are included in the article/**Supplementary material**, further inquiries can be directed to the corresponding author.
