# Supplementary material for: Association of volatile anesthesia exposure and depth with emergence agitation and delirium in children: Prospective observational cohort study
Source: Front Pediatr. 2023 Mar 23;11:1115124. doi: 10.3389/fped.2023.1115124 (PMC10076635; doi:10.3389/fped.2023.1115124)
Supplement: Supplementary file 1 [file Table1.docx]

| **Supplemental table 1. Comparison between emergence agitation and emergence delirium outcomes** | | | | | | | | |
| --- | --- | --- | --- | --- | --- | --- | --- | --- |
|  | **Emergence agitation** | | | | **Emergence delirium** | | | |
|  | No EA | EA | Effect Estimate | *P* Value | No ED | ED | Effect Estimate | *P* Value |
|  | n=64 | n=73 |  |  | n=62 | n=75 |  |  |
| Age (y) | 9.00 (7.00, 11.00) | 9.00 (8.00, 10.00) | 0.103 | 0.918 | 9.00 (8.00, 11.00) | 9.00 (8.00, 10.00) | 0.092 | 0.926 |
| Weight (kg) | 26.63 (28.2, 59.7) | 27.16 (24.9, 61.8) | 0.091 | 0.933 | 27.41 (21.6, 65.8) | 27.63 (26.8, 66.1) | 0.101 | 0.922 |
| BMI (kg/m^2^) | 16.3 (14.1, 21.4) | 16.6 (13.9, 20.5) | 0.105 | 0.921 | 15.9 (13.8, 20.1) | 16.2 (14.2, 21.3) | 0.098 | 0.903 |
| BIS, N [%] |  |  |  |  |  |  |  |  |
| BIS＞40 | 28 (43.8%) | 36 (56.3%) | 0.227 | 0.634 | 25 (40.3%) | 37 (59.7%) | 0.077 | 0.782 |
| BIS≤40 | 29 (39.7%) | 44 (60.3%) |  |  | 32(42.7%) | 43 (57.3%) |  |  |
| TimeLOW-BIS/Time_anes_ %,  N [%] |  |  |  |  |  |  |  |  |
| TimeBIS≤40/Time_anes_ % | 80.5%  (60.0%, 95.0%) | 85.0%  (62.0%, 98.5%) | -0.261 | 0.794 | 88.0%  (64.3%, 99.0%) | 85.0%  (53.0%, 94.0%) | -1.002 | 0.317 |
| TimeBIS≤30/Time_anes_ % | 34.5%  (19.3%, 68.0%) | 36.0%  (14.0%, 59.0%) | -0.252 | 0.801 | 41.0%  (19.0%, 69.3%) | 29.0%  (11.0%, 55.0%) | -1.543 | 0.123 |
| TimeBIS≤20/Time_anes_ % | 10%  (3.3%, 16.0%) | 9%  (1.0%, 20.0%) | -0.827 | 0.408 | 10%  (2.3%, 18.3%) | 9%  (2.0%, 18.0%) | -0.211 | 0.833 |
| EtSevo-time AUC | 2551.00 (2249.00, 3058.00) | 2740.00 (2450.00, 3173.00) | 2.282 | 0.022 | 2571.00 (2268.50, 3054.75) | 2740.00 (2438.00, 3164.00) | 1.834 | 0.067 |
| EtSevo (%) | 4.55 (4.00, 4.86) | 4.43 (4.10, 4.93) | 0.298 | 0.766 | 4.25 (4.00, 4.77) | 4.56 (4.10, 4.93) | 1.332 | 0.183 |
| m-YPAS | 28.13 (22.92, 33.33) | 29.17 (22.92, 39.58) | 0.660 | 0.509 | 28.13 (22.92, 33.89) | 29.92 (29.17, 35.42) | 0.514 | 0.607 |
| Intraoperative agitation,  N [%] |  |  |  |  |  |  |  |  |
| No | 40 (62.5%) | 24 (37.5%) | 6.252 | 0.012 | 41 (66.1%) | 21 (33.9%) | 10.244 | 0.001 |
| Yes | 30 (41.1%) | 43 (58.9%) |  |  | 28 (38.7%) | 46 (61.3%) |  |  |
| Accident  (Respiratory complications), N [%] |  |  |  |  |  |  |  |  |
| No | 55(85.9%) | 9 (14.1%) | 0.662 | 0.416 | 58 (93.5%) | 4 (6.5%) | 3.000 | 0.083 |
| Yes | 66(90.4%) | 7 (9.6%) |  |  | 63 (84.0%) | 12 (16.0%) |  |  |
| Anesthesia time (min) | 9.00 (8.00, 11.00) | 10.00 (8.00, 12.00) | 1.564 | 0.118 | 9.00 (8.00, 11.00) | 10.00 (8.00, 11.00) | 0.688 | 0.491 |
| Surgical time (min) | 5.00 (4.00, 5.00) | 5.00 (5.00, 6.00) | 3.347 | 0.001 | 5.00 (5.00, 6.00) | 5.00 (5.00, 6.00) | 0.415 | 0.678 |
| Peak FLACC | 1.50 (1.00, 3.00) | 3.00 (3.00, 5.00) | 6.397 | ＜0.001 | 1.50 (1.00, 3.00) | 3.00 (3.00, 5.00) | 6.666 | ＜0.001 |

Values are presented as Median (25%, 75% Quartile), N [%]. Abbreviations: BMI, body mass index; ASA, American Society of Anesthesiologists; m-YPAS, modified Yale preoperative anxiety scale; BIS, bispectral index; EtSevo, end-tidal sevoflurane concentration; AUC, the area under curve; Peak FLACC, the peak scores of face, legs, activity, cry, and consolability.
