# Supplementary material for: Association of volatile anesthesia exposure and depth with emergence agitation and delirium in children: Prospective observational cohort study
Source: Front Pediatr. 2023 Mar 23;11:1115124. doi: 10.3389/fped.2023.1115124 (PMC10076635; doi:10.3389/fped.2023.1115124)
Supplement: Supplementary file 2 [file Table2.docx]

| **Supplemental table 2.** **Crude odds ratio for emergence agitation and emergence delirium** | | | | | |  |
| --- | --- | --- | --- | --- | --- | --- |
|  | | **Emergence agitation** |  | **Emergence delirium** | |  |
|  |  |  |  |  |  |  |
|  |  |  |  |  |  |  |
| **Variable** |  | **Odds Ratio (95%CI)** | **p value** | **Odds Ratio (95%CI)** | **p value** |  |
|  |  |  |  |  |  |  |
|  |  |  |  |  |  |  |
| Age |  | 1.00 (0.83, 1.21） | 1.000 | 1.03 (0.85, 1.25） | 0.770 |  |
| EtSevo-time AUC |  | 1.00 (1.00, 1.00） | 0.001 | 1.00 (1.00, 1.00） | 0.066 |  |
| EtSevo-time AUC | ≤2000 | Reference |  | Reference |  |  |
|  | 2000-2500 | 3.08 (0.59, 16.11） | 0.183 | 1.00 (0.26, 3.93） | 1.000 |  |
|  | 2500-3000 | 7.33 (1.34, 40.21） | 0.022 | 1.27 (0.31, 5.20） | 0.743 |  |
|  | ＞3000 | 6.44 (1.23～33.80） | 0.028 | 1.47 (0.38, 5.80） | 0.579 |  |
| EtSevo |  | 1.03 (0.60, 1.79） | 0.907 | 1.30 (0.75, 2.26） | 0.353 |  |
| m-YPAS |  | 1.01 (0.97,1.04） | 0.672 | 1.00 (0.97, 1.04） | 0.842 |  |
| Anesthesia time |  | 1.15 (0.97, 1.35） | 0.106 | 1.10 (0.94, 1.30） | 0.238 |  |
| Surgical time |  | 1.64 (1.16, 2.31） | 0.005 | 1.14 (0.89, 1.47） | 0.305 |  |
| Peak FLACC |  | 1.94 (1.43, 2.64） | 0.000 | 2.89 (1.93, 4.33） | 0.000 |  |
| BIS | BIS＞40 | Reference |  | Reference |  |  |
|  | BIS≤40 | 0.93 (0.47, 1.83） | 0.827 | 0.80 (0.41, 1.59） | 0.532 |  |
| Intraoperative agitation | No | Reference |  | Reference |  |  |
|  | Yes | 0.73 (0.37, 1.43） | 0.355 | 1.17 (0.60, 2.29） | 0.650 |  |
| Accident (Respiratory complications) | No | Reference |  | Reference |  |  |
|  | Yes | 0.65 (0.23, 1.85） | 0.419 | 0.81 (0.28, 2.29） | 0.685 |  |

Abbreviations: BIS, bispectral index; EtSevo, end-tidal sevoflurane concentration; AUC, the area under curve; m-YPAS, modified Yale preoperative anxiety scale; Peak FLACC, the peak scores of face, legs, activity, cry, and consolability.
