# Supplementary figures and images for: Association of volatile anesthesia exposure and depth with emergence agitation and delirium in children: Prospective observational cohort study
Source: Front Pediatr. 2023 Mar 23;11:1115124. doi: 10.3389/fped.2023.1115124 (PMC10076635; doi:10.3389/fped.2023.1115124)

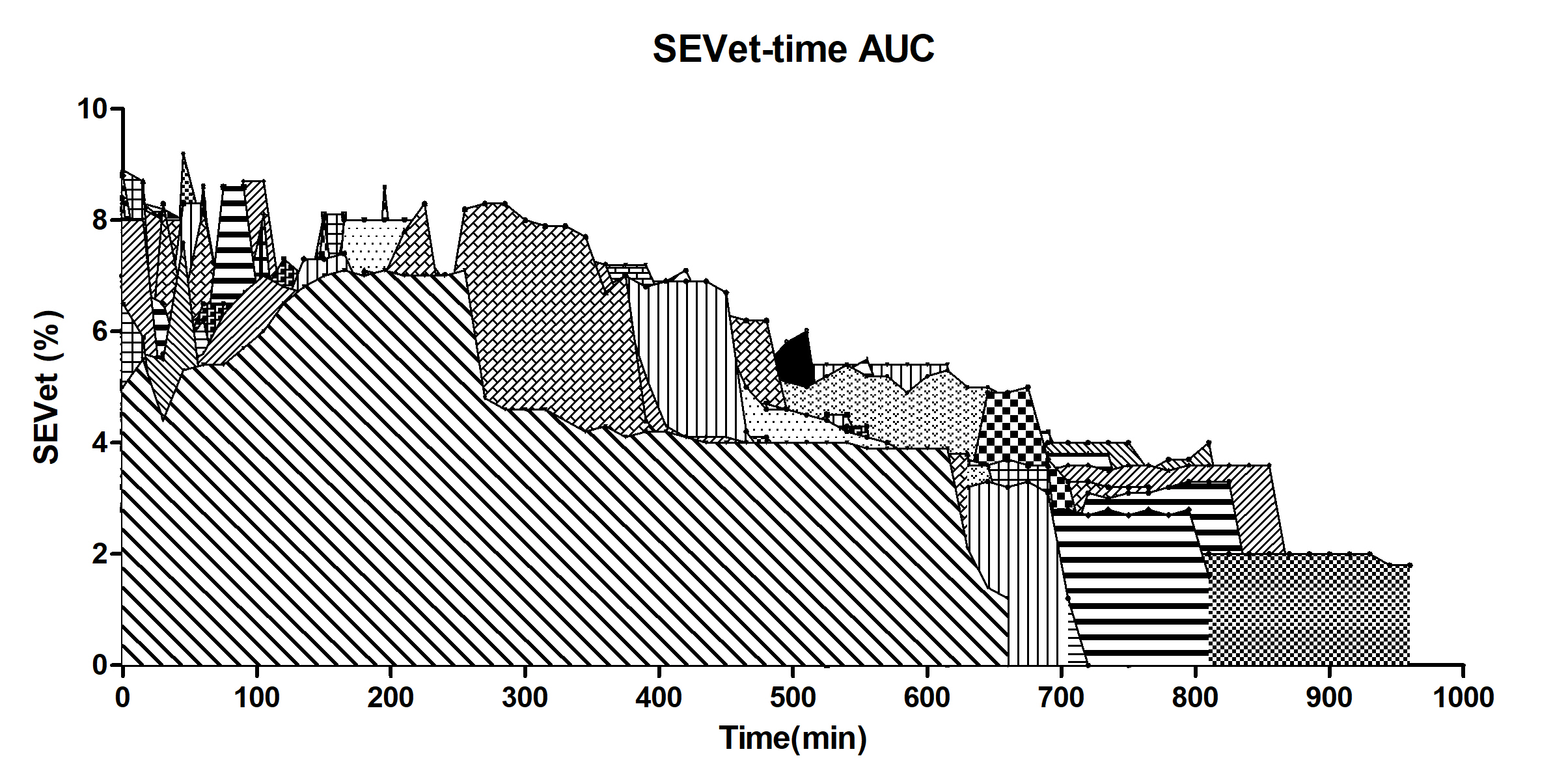

Supplement: Supplementary file 3 [file Image1.jpeg]
